# Metabolomics Provides New Insights into Host Manipulation Strategies by *Asobara japonica* (Hymenoptera: Braconidae), a Fruit Fly Parasitoid

**DOI:** 10.3390/metabo13030336

**Published:** 2023-02-24

**Authors:** Shengmei Liu, Junwei Zhang, Yifeng Sheng, Ting Feng, Wenqi Shi, Yueqi Lu, Xueying Guan, Xuexin Chen, Jianhua Huang, Jiani Chen

**Affiliations:** 1 Institute of Insect Sciences, Ministry of Agriculture Key Lab of Molecular Biology of Crop Pathogens and Insect Pests, Zhejiang University, Hangzhou 310058, China; 2 Key Laboratory of Biology of Crop Pathogens and Insects of Zhejiang Province, Zhejiang University, Hangzhou 310058, China; 3 Zhejiang Provincial Key Laboratory of Crop Genetic Resources, Institute of Crop Science, Plant Precision Breeding Academy, College of Agriculture and Biotechnology, Zhejiang University, Hangzhou 310058, China

**Keywords:** parasitoid wasp, parasitization, metabolomics, metabolite, host manipulation

## Abstract

*Asobara japonica* (Hymenoptera: Braconidae) is an endoparasitoid wasp that can successfully parasitize a wide range of host species across the *Drosophila* genus, including the invasive crop pest *Drosophila suzukii*. Parasitoids are capable of regulating the host metabolism to produce the nutritional metabolites for the survival of their offspring. Here, we intend to investigate the metabolic changes in *D. melanogaster* hosts after parasitization by *A. japonica*, using the non-targeted LC-MS (liquid chromatography-mass spectrometry) metabolomics analysis. In total, 3043 metabolites were identified, most of which were not affected by *A. japonica* parasitization. About 205 metabolites were significantly affected in parasitized hosts in comparison to non-parasitized hosts. The changed metabolites were divided into 10 distinct biochemical groups. Among them, most of the lipid metabolic substances were significantly decreased in parasitized hosts. On the contrary, most of metabolites associated with the metabolism of amino acids and sugars showed a higher abundance of parasitized hosts, and were enriched for a wide range of pathways. In addition, eight neuromodulatory-related substances were upregulated in hosts post *A. japonica* parasitization. Our results reveal that the metabolites are greatly changed in parasitized hosts, which might help uncover the underlying mechanisms of host manipulation that will advance our understanding of host–parasitoid coevolution.

## 1. Introduction

Parasitoid wasps are a large group of hymenopteran insects, comprising an estimated number of 150,000 to 600,000 species [1]. They are free-living insects as adults; however, their wasp larvae must parasitize the various life stages of other arthropods, referred to as hosts [2,3]. The female wasps lay eggs in (endoparasitoids) or on (ectoparasitoids) the host body, following the hatched wasp larvae consuming and finally killing their hosts [1,4]. Consequently, parasitoids are ideal biocontrol agents to constrain the destructive pest populations in agriculture ecosystems [5,6,7].

Most of the endoparasitoids belong to koinobionts, whose hosts are allowed to continue growing post parasitism, and thus provide enough nutrients for wasp offspring development and survival [1]. As such, the endoparasitoids have evolved many effective strategies to escape or suppress the host immune response [5,6,8]. Moreover, successful parasitization also requires the regulation of host nutritional metabolism to allow the wasp eggs to hatch and wasp larvae to develop [9,10,11].

Different with insect predators that attain nutrients through external preys, the endoparasitoid species typically rely on the resources derived from a single host to complete their immature development, and usually fails to synthetize some essential nutrients (e.g., lipids) by themselves [9,12,13]. So far, some endoparasitoids have been reported to manipulate the nutrients of their hosts post parasitization. For instance, quantities of free amino acids and proteins were varied in *Devorgilla canescens*-infected *Anagasta kuehniella* hosts [14]; the contents of total lipids and proteins were decreased and the contents of glycogen were increased in parasitized *Lymantria dispar* [15]; and the contents of proteins were decreased and the contents of lipids were increased in *Spodoptera littoralis* after parasitization by *Chelonus inanitus* [16]. Recently, results from *Leptopilina boulardi*, an endoparasitoid wasp of the *Drosophila* species, showed that parasitization reduced the lipid content in host hemolymph, but lipids were accumulated in host body fat cells [9,17]. Further functional analysis revealed that the lipid nutrients were necessary for the survival of parasitoid offspring [9,17,18]. Collectively, these findings propose that the parasitoids have the ability to regulate the composition of host nutrients to meet the dietary demands of their progeny.

Nutritional metabolites participate in various pathways which are associated with insect development and reproduction. Over the last decade, metabolomics has become one of the most advanced technologies in life science studies, and this technique has also been greatly applied in insects [19], including liquid chromatography-mass spectrometry (LC-MS) [20], gas chromatography-mass spectrometry (GC-MS) [21] and nuclear magnetic resonance (NMR) [22]. Based on high-throughput analysis, the changes of metabolites and the relevant metabolic processes have been discovered in some insect species that were either experiencing different environmental stresses [23,24,25], or associated with plant–insect interactions [26,27,28,29]. However, the metabolomics analyses in parasitoid wasps, especially for dissecting host nutritional manipulation strategies, are largely unexplored.

*Asobara japonica* (Hymenoptera: Braconidae) is a highly generalized larval-pupal endoparasitoid, that parasitizes a number of host species across the *Drosophila* genus, thus being considered as a model organism for exploring the mechanisms under host–parasitoid interactions [30,31]. Here we applied liquid chromatography-mass spectrometry (LC-MS)-based metabolomics technology to analyze the metabolic changes in identify main metabolic pathways altered by *A. japonica* parasitization in *D. melanogaster* hosts. Our data will therefore contribute to the understanding of the host manipulation strategies and provide a new perspective on host–parasitoid coevolution.

## 2. Materials and Methods

### 2.1. Insects

The parasitoid *A. japonica* was collected from Taizhou (28°50′ N, 120°34′ E), Zhejiang, China, in June 2018 [32]. Since then, this strain was maintained in our lab on *D. melanogaster W^1118^* as a regular host, which was raised on standard cornmeal medium at 25 °C, 50% humidity and 16 L:8 D inside plastic fly bottles. The newly emerged adult wasps were provided with an apple juice agar medium until exposure to hosts. The apple juice agar recipe: 27 g agar, 33 g brown sugar and 330 mL pure apple juice diluted in 1000 mL of water [33].

### 2.2. Experimental Procedures and Sample Collection

**Figure 1:**
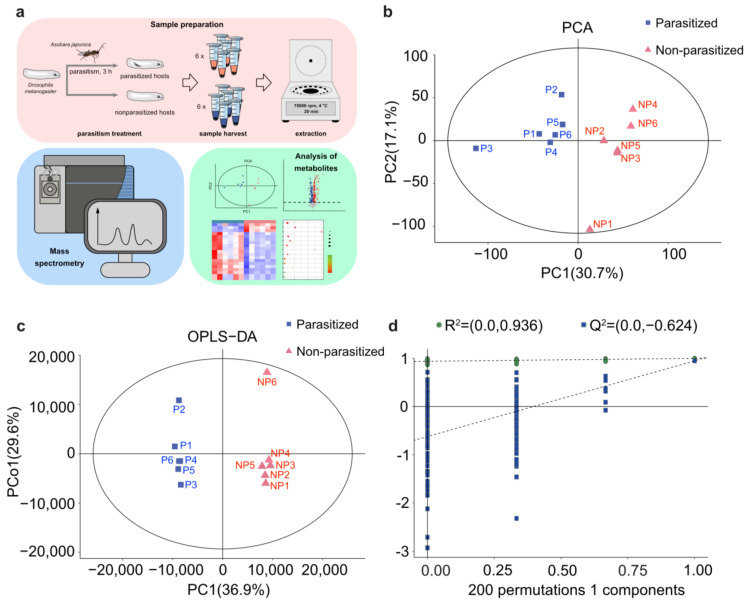
PCA and OPLS-DA analyses of metabolic differences. (**a**) Workflow for metabolomics analysis. (**b**) Principal component (PC) analysis (PCA) score plot of the metabolite profiles of *D. melanogaster* larvae in the parasitized group and non-parasitized group. Data points are displayed as sample projections on the two primary axes. The oval represents the 95% confidence interval of the model using Hotelling’s T2 statistics. Variances explained by the first two components (PC1 and PC2) are shown in parentheses. (**c**) Orthogonal partial least-squares discriminant analysis (OPLS-DA) score plot shows a clear separation of metabolic profiles between the parasitized and non-parasitized *D. melanogaster* larvae. Variances explained by the principal components (PC1 and PCo1) are shown in parentheses. Abbreviations: PC1, predictive component 1; PCo1, predictive component orthogonal 1; NP, non-parasitized host; P, parasitized host. (**d**) Permutation tests of OPLS-DA models. The permutation test was carried out with 200 random permutations, and the resulting R^2^ and Q^2^ values were plotted. Green circle, R^2^; Blue square, Q^2^.

Three-day-old *A. japonica* females were used to parasitize the 2nd instar *Drosophila* host larvae for 3 h, to ensure that approximately all the hosts were parasitized. The number of female wasps was based on the parasitoid/host ratio of ~1:10. The *Drosophila* host larvae were collected after 3 h parasitization, along with the non-parasitized hosts at the same age (control), washed in 75% alcohol, and rinsed with ddH_2_O 3 times. After air-drying, the *Drosophila* host larvae were moved to tubes containing 150 μL of 80% methanol for grinding, and then centrifuged at 15,000 rpm for 20 min at 4 °C. The supernatant was extracted into 1.5 mL centrifuge tubes, frozen in liquid nitrogen, and stored at −80 °C for further use. In this study, we collected two different groups, including *A. japonica*-parasitized and non-parasitized *Drosophila* host larvae, for metabolomics analysis, and analysis of each group was repeated six times. Approximately 130 host larvae were used in each repetition (Figure 1a).

### 2.3. Sample Pretreatment

Samples were pretreated as described previously, with minor modifications [34,35]. Briefly, a total of 50 μL supernatant from each sample was transferred into a 1.5 mL microcentrifuge tube containing 20 μL internal standard buffer (L-2-chlorophenylalanine in methanol, 0.06 mg/mL), and vortexed for 10 s. Then, 200 μL protein precipitant of methanol: acetonitrile at 2:1 ratio (*v*/*v*) was added to each sample and vortexed for 1 min, followed by ultrasonicating in an ice-water bath for 10 min, and placed at −20 °C for 30 min. Treated samples were centrifuged at 13,000 rpm at 4 °C for 10 min, and all supernatants were extracted into individual vials for volatilization. Thereafter, 200 μL methanol–water solution at 1:4 ratio (*v*/*v*) was added to the dried samples for dissolution. The samples were vortexed for 30 s, ultrasonicated for 3 min, placed at −20 °C for 2 h, and then centrifuged at 13,000 rpm at 4 °C for 10 min. Finally, a supernatant of 150 μL from each sample was filtered through 0.22 μm microfilters and stored at −80 °C for metabolomics analysis (OE Biotech, Shanghai, China).

### 2.4. LC-MS/MS Analysis

Nontargeted LC-MS analyses were performed using the UPLC-Q-Exactive Orbitrap/MS (Shimadzu Corporation, Kyoto, Japan) platform, which was equipped with a heated electrospray ionization (ESI) source (Thermo Fisher Scientific, Waltham, MA, USA), to analyze the metabolic profiling in both ESI-positive and ESI-negative ion modes. An ACQUITY UPLC HSS T3 column (2.1 mm × 100 mm, 1.8 μm), with a maintained temperature of 45 °C, was applied in both positive and negative modes. Mobile phases consisted of 0.1% formic acid in water (A) and acetonitrile (B), and the gradient elution conditions were as follows: 0 min, 5% B; 2 min, 5% B; 4 min, 30% B; 8 min, 50% B; 10 min, 80% B; 14 min, 100% B; 15 min, 100% B; 15.1 min, 5% B; and 16 min, 5% B. The flow rate was 0.35 mL/min, and the injection volume was 2 μL.

The mass range was set at m/z 125–1000. The resolution of the full scan was set to 70,000 and 17,500 for HCD MS/MS. When operating the mass spectrometer, the spray voltage was set to 3500 V for positive or 3500 V for negative; sheath gas-flow rate was set to 40 arbitrary units for positive or 35 arbitrary units for negative; auxiliary gas-flow rate was set to 10 arbitrary units for positive or 8 arbitrary units for negative; capillary temperature was set to 320 °C. For MS/MS analysis, the collision energy was set to 10, 20 and 40 eV.

### 2.5. Data Analysis

The LC-MS raw data were analyzed using the Progenesis QI software V2.3 (Nonlinear, Newcastle upon Tyne, UK). Precursor tolerance was set at 5 ppm, product tolerance was set at 10 ppm, and product ion threshold was set at 5%. Metabolites were identified based on precise mass-to-charge ratio (m/z), secondary fragments, and isotopic distribution. Public databases, such as human metabolome database (HMDB, http://www.hmdb.ca/, accessed on 10 December 2022); Lipidmaps (v2.3, http://www.lipidmaps.org/, accessed on 1 June 2022) and Metlin (https://metlin.scripps.edu/, accessed on 1 June 2022), were used for qualitative analysis.

The LC-MS raw data were imported into R package for multivariate statistical analysis, such as principal component analysis (PCA) and orthogonal partial least-squares discriminant analysis (OPLS-DA). PCA was carried out to initially visualize the overall distribution among the samples and the stability of the whole analysis process. OPLS-DA was used to distinguish the metabolites that differ between parasitized and non-parasitized control groups. Variable importance in the projection (VIP) was obtained from OPLS-DA to rank the overall contribution of each variable item to group discrimination. The univariate analysis used *t*-tests, and fold-change (FC) analysis was also used to compare the metabolites between the two groups. FC was calculated as the ratio of the peak area of parasitized and non-parasitized groups. Additionally, the screening criteria for differential metabolites was the VIP values from the OPLS-DA model > 1 and the *p*-value from the Student’s *t*-test < 0.05, based on the previous reports [36,37,38]. Here, the default 7-fold cross-validation was applied with 1/7 of the samples being excluded from the mathematical model in each round, to guard against overfitting.

Differential metabolites were mapped onto the Kyoto Encyclopedia of Genes and Genomes (KEGG) database, to acquire detailed pathway information. The KEGG metabolic pathways were then predicted from the differential metabolites showing a *p*-value < 0.05. Statistical analyses were performed in GraphPad Prism version 9.0 (GraphPad Software).

## 3. Results

### 3.1. PCA and OPLS-DA Analyses of Metabolic Difference in Parasitized Host

To comprehensively analyze the profiles of metabolites, nontargeted LC-MS was performed in *A. japonica*-parasitized and non-parasitized *Drosophila* host larvae (Figure 1a). The PCA analysis showed a clear separation between the *A. japonica*-parasitized and non-parasitized samples, and they were all within the 95% confidence interval of the Hotelling’s T2 ellipse (Figure 1b). The results indicated that no outlier was present, and all samples were suitable for the following analysis. Meanwhile, the OPLS-DA model showed that the parasitized samples were clustered on the left part of the x-axis, and the non-parasitized samples were clustered on the right (Figure 1c), revealing that significant differences existed between the two groups. Seven-fold cross validation and 200 response permutation testing (RPT) was then performed in the OPLS-DA model. The resulting R^2^ was 0.936 and Q^2^ was −0.624 (Figure 1d), reflecting that the OPLS-DA did not exceed the fit. The explanatory ability (R^2^Y(CUM)) and the predictive ability (Q^2^(CUM)) for the sample were 0.997 and 0.946, respectively, suggesting that the established model conformed to the real situation of the data. These results indicate that the metabolites of *D. melanogaster* hosts were greatly changed after parasitization by *A. japonica*.

### 3.2. Changes in Metabolites Post A. japonica Parasitization

**Figure 2:**
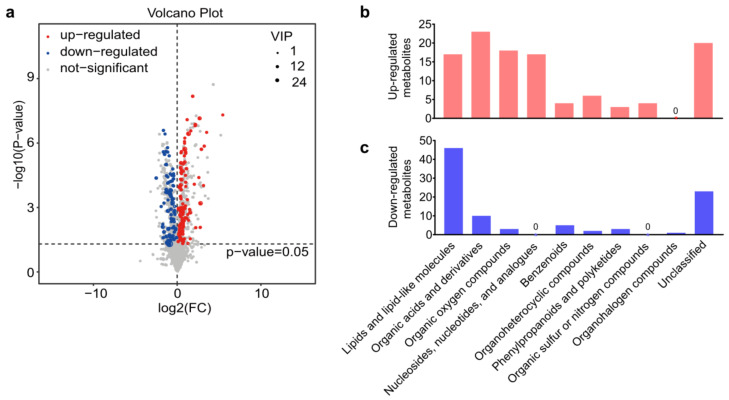
Differential metabolites between parasitized and non-parasitized hosts. (**a**) Volcano plot of the 205 differential metabolites. *Each point in the volcano diagram represents a metabolite, and the greater the scattered point, the greater the value of variable importance is in the projection (VIP).* (**b**) The classification of upregulated metabolites in parasitized hosts. (**c**) The classification of downregulated metabolites in parasitized hosts. Abbreviations: FC, fold change; VIP, variable important in projection.

A total of 8563 peaks were detected by LC-MS, including 3339 peaks from the negative ion model and 5224 peaks from the positive ion model, respectively. In addition, 3043 metabolites that consisted of 868 negative ion mode metabolites (ESI−) and 2175 positive ion mode metabolites (ESI+) were identified with annotations. After the exogenous compounds in LC-MS were removed, the differentially abundant metabolites were selected according to the VIP values from the OPLS-DA model (VIP > 1) and the *p*-value from the Student’s *t*-test (*p* < 0.05). Volcano plots showed all the 205 differentially expressed metabolites that identified between *A. japonica*-parasitized and non-parasitized *D. melanogaster* hosts (Figure 2a), including 112 upregulated metabolites and 93 downregulated metabolites (Table S1). Those differential metabolites were divided into ten distinct biochemical groups (Figure 2b,c): lipids and lipid-like molecules (63/205, 30.73%); organic acids and derivatives (33/205, 16.10%); organic oxygen compounds (21/205, 10.24%); nucleosides, nucleotides, and analogues (17/205, 8.29%); benzenoids (9/205, 4.39%); organoheterocyclic compounds (8/205, 3.90%); phenylpropanoids and polyketides (6/205, 2.93%); organic sulfur or nitrogen compounds (4/205, 1.95%); organohalogen compounds (1/205, 0.49%) and unclassified metabolites (43/205, 20.98%). We next mainly focused on the top three groups of differential metabolites.

### 3.3. Differences in Lipids and Lipid-like Molecules Post A. japonica Parasitization

**Figure 3:**
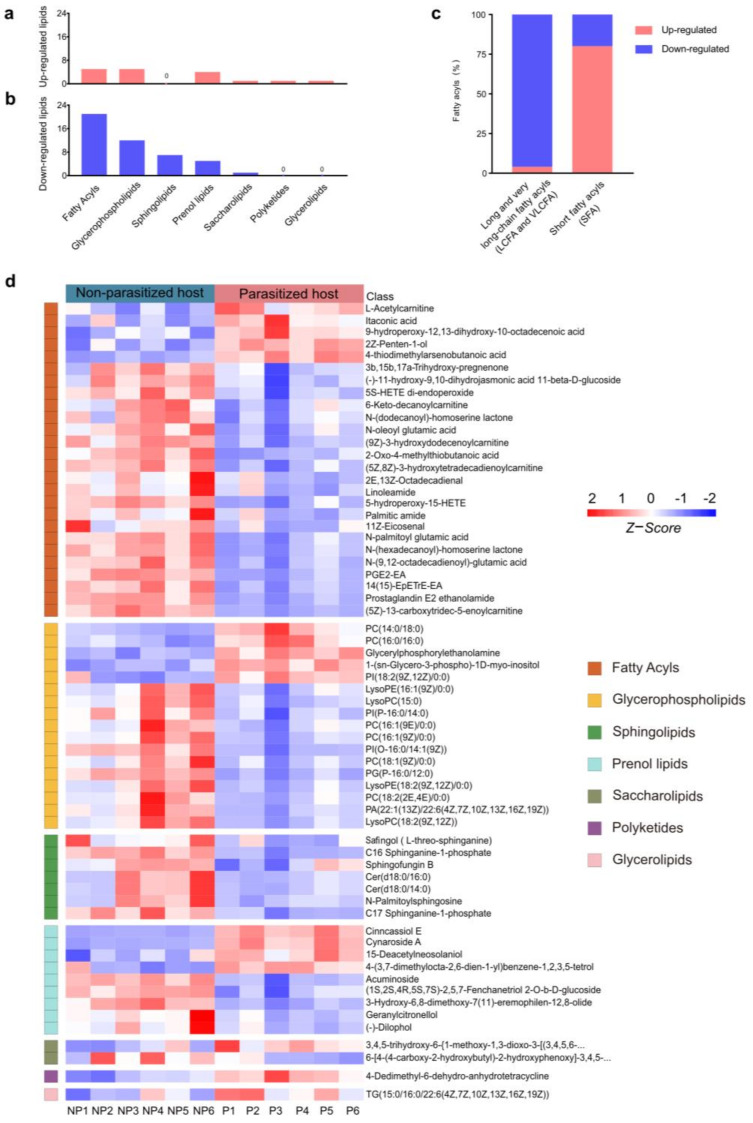
Differences in lipids and lipid-like molecules post *A. japonica* parasitization. (**a**) The classification of upregulated lipids and lipid-like molecules. (**b**) The classification of downregulated lipids and lipid-like molecules. (**c**) Distribution of differential long-chain fatty acyls (LCFAs) or very long-chain fatty acyls (VLCFAs) and short fatty acyls (SFAs) in the classification of fatty acyls (FAs). (**d**) Heatmap for comparison of lipids and lipid-like molecules between parasitized and non-parasitized hosts. *Each column represents one NP or P sample, and each row represents one differentially expressed metabolite. The Z-score of each sample is plotted according to the red–blue color scale. The red and blue colors indicate high abundance and low abundance, respectively.* Abbreviations: NP, non-parasitized host; P, parasitized host.

The most altered group of metabolites was the lipids and lipid-like molecules, which can be divided into fatty acyls (FA), glycerophospholipids (GP), sphingolipids (SP), prenol lipids (PR), saccharolipids (SL), polyketides (PK) and glycerolipids (GL), according to different structures and synthetic pathways [39] (Figure 3a,b,d). In this group, a total of 63 differential metabolites were identified, most (46/63, 73.02%) of which were downregulated in parasitized hosts, including FA (21/46, 45.65%), GP (12/46, 26.09%), SP (7/46, 15.22%), PR (5/46, 10.87%) and SL (1/46, 2.17%) (Table S1 and Figure 3a,b). Generally, FA containing more than 13 carbon atoms is referred to as a long-chain fatty acyl (C: 13-21, LCFA) or very long-chain fatty acyl (C > 21, VLCFA), while the FA containing less than 13 carbon atoms is determined as short fatty acyl (SFA) [40]. Among the total of five upregulated FAs and 21 downregulated FAs, LCFAs and VLCFAs accounted for a large proportion (21/26), and almost all were declined in parasitized hosts (20/21, Figure 3c). As for the remaining five differentially expressed SFAs (L-Acetylcarnitine, Itaconic acid, 2Z-Penten-1-ol, 4-thiodimethylarsenobutanoic acid, 2-Oxo-4-methylthiobutanoic acid), there was only one decreased substance (*2-oxo-4-methylthiobutanoate*) (Figure 3c). Though most of the lipids and lipid-like molecules were decreased, the cluster heatmap showed that parasitization increased some metabolites in six out of the seven groups, except in SP, where the metabolites were all decreased (Figure 3d).

### 3.4. Differences in Organic Acids and Their Derivatives Post A. japonica Parasitization

**Figure 4:**
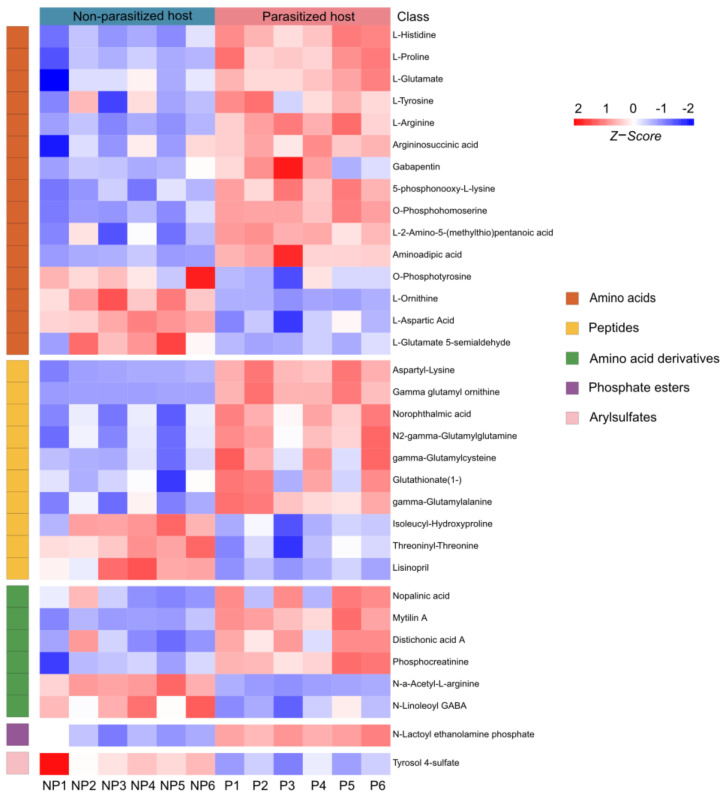
Differences in organic acids and derivatives post *A. japonica* parasitization. Heatmap of the group of organic acids and derivatives changing in parasitized and non-parasitized hosts. Each column represents one NP or P sample, and each row represents one differentially expressed metabolite. The Z-score of each sample is plotted according to the red–blue color scale. The red and blue colors indicate high abundance and low abundance, respectively. Abbreviations: NP, non-parasitized host; P, parasitized host.

A total of 33 differential metabolites of organic acids and derivatives were annotated, including the classes of amino acids (15/33, 45.45%), peptides (10/33, 30.30%), amino acid derivatives (6/33, 18.18%), phosphate esters (1/33, 3.03%) and arylsulfates (1/33, 3.03%) (Table S1 and Figure 4). Amino acids are necessary in the nutritional and metabolic processes of insects. It has been reported that amino acids from hosts are essential for the hatching of parasitoid eggs [41]. In our study, the majority of amino acids (11/15, 73.33%) were increased, except O-phosphotyrosine, L-ornithine, L-aspartic acid and L-glutamate 5-semialdehyde (Figure 4). Moreover, we also found 10 peptides of which the expression changed post parasitization, with three of them (isoleucyl-hydroxyproline, threoninyl-threonine and lisinopril) being downregulated and seven (aspartyl-lysine, gamma glutamyl ornithine, norophthalmic acid, N2-gamma-glutamylglutamine, gamma-glutamylcysteine, glutathionate(1-), and gamma-glutamylalanine) being upregulated (Figure 4). As for the class of the amino acid derivatives, parasitization significantly increased the contents of four substances (nopalinic acid, mytilin A, distichonic acid A and phosphocreatinine), while decreasing those of the remaining two substances (N-a-acetyl-L-arginine and N-Linoleoyl GABA) in parasitized hosts (Figure 4). Compared with the healthy *Drosophila* hosts, the metabolite of phosphate ester (N-lactoyl ethanolamine phosphate) was increased, while the arylsulfate (tyrosol 4-sulfate) was decreased after parasitization (Figure 4).

### 3.5. Changes in Organic Oxygen Compounds Post A. japonica Parasitization

**Figure 5:**
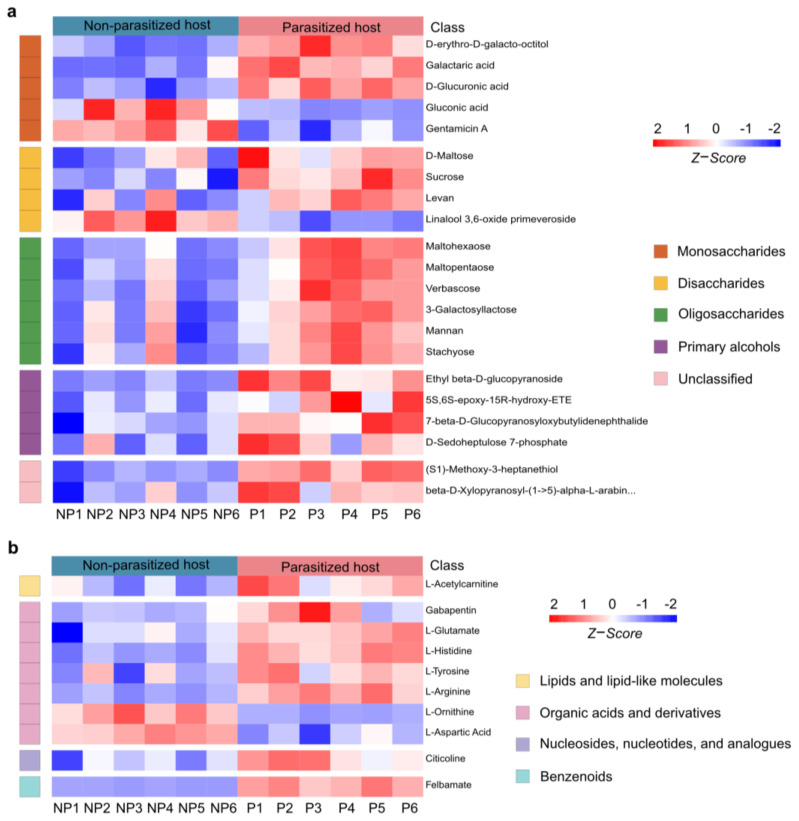
Changes in organic oxygen compounds and neuromodulators post *A. japonica* parasitization. (**a**) Heatmap of the groups of organic oxygen compounds changing in parasitized and non-parasitized hosts. (**b**) Heatmap for comparison of neuromodulators between parasitized and non-parasitized hosts. Each column represents one NP or P sample, and each row represents one differentially expressed metabolite. The Z-score of each sample is plotted according to the red–blue color scale. The red and blue colors indicate high abundance and low abundance, respectively. Abbreviations: NP, non-parasitized host; P, parasitized host.

The differential organic oxygen compounds detected in *A. japonica*-parasitized and non-parasitized hosts have been further divided into monosaccharides, disaccharides, oligosaccharides, primary alcohols and other metabolites (Figure 5a), which came to a total of 21 metabolites. As shown in Figure 5a, parasitization significantly unregulated most (18/21, 85.71%) of the organic oxygen compounds in hosts, while two monosaccharides (gluconic acid and gentamicin A) and one disaccharide (linalool 3,6-oxide primeveroside) were largely downregulated. Interestingly, different types of organic oxygen compounds were found to be involved in the immune responses. For examples, levan, an increased disaccharide, has been used as an anti-irritant, anti-oxidant and anti-inflammatory agent [42]; mannan, an increased oligosaccharide, can function as an immunosuppressant in response to fungus infection [43]; another upregulated oligosaccharide, stachyose, is able to increase both glutathione peroxidase and superoxide dismutase activities, which could remarkably decrease the inflammation response [44]. Taken together, these results suggest that the changed carbohydrate metabolites not only provide the necessary carbon and energy sources for parasitoid offspring, but also might help increase host immunity.

### 3.6. Changes in Neuromodulators Post A. japonica Parasitization

Neuromodulators are an important type of metabolite, which is widely present in insects and mainly controls their numerous behaviors. As a functional active ingredient, neuromodulators have attracted extensive attention in parasitoid–host manipulation [45,46,47,48]. Compared to the non-parasitized hosts, a total of 10 neuromodulators were identified to be changed in *A. japonica*-parasitized hosts. They were from the groups of lipids and lipid-like molecules (1/10, 10%), organic acids and derivatives (7/10, 70%), nucleosides, nucleotides, and analogues (1/10, 10%) and benzenoids (1/10, 10%) (Figure 5b). Organic acids and derivatives were the most abundant neuromodulators among the four biochemical groups, with five of them (gabapentin, L-glutamate L-histidine L-tyrosine L-arginine) being upregulated and two of them (L-ornithine and L-aspartic Acid) being downregulated. The remaining three differential neuromodulators in the group of lipids and lipid-like molecules (L-acetylcarnitine), nucleosides, nucleotides, and analogues (citicoline), and benzenoids (felbamate) were significantly increased after parasitization. Remarkably, felbamate, a nerve suppressor and antiseizure drugs (ASDs), was the most upregulated neuromodulator upon *A. japonica* infection. (42.47-fold, Table S1).

### 3.7. Metabolic Pathway Analysis

**Figure 6:**
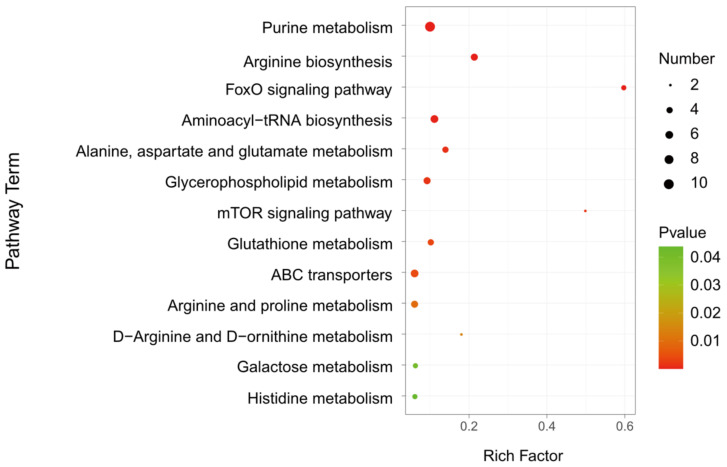
KEGG analysis of the differential metabolites in *D. melanogaster* hosts post *A. japonica* parasitization. KEGG analysis of differential metabolites in parasitized and non-parasitized hosts. The larger the rich factor, the greater the enrichment degree; the color from green to red indicates that the *p*-value decreasing. The larger the point, the more metabolites enriched in the pathway.

To identify the metabolic pathways altered in parasitized hosts, 205 differential metabolites were further carried out by the KEGG analysis. A total of 66 differential metabolites were identified, which were distributed in 47 metabolic pathways (Table S2). Taking a *p*-value ≤ 0.05 as the threshold, 13 pathways satisfying the condition were significantly enriched by different metabolites (Figure 6 and Table 1), including purine metabolism (KEGG: dme00230; *p* = 2.51 × 10^−6^), arginine biosynthesis (KEGG: dme00220; *p* = 3 × 10^−5^), FoxO signaling pathway (KEGG: dme04068; *p* = 4.44 × 10^−5^), aminoacyl-tRNA biosynthesis (KEGG: dme00970; *p* = 0.00019), alanine, aspartate and glutamate metabolism (KEGG: dme00250; *p* = 0.00110), glycerophospholipid metabolism (KEGG: dme00564; *p* = 0.00160), mTOR signaling pathway (KEGG: dme04150; *p* = 0.00165), glutathione metabolism (KEGG: dme00480; *p* = 0.00349), ABC transporters (KEGG: dme02010; *p* = 0.00426), arginine and proline metabolism (KEGG: dme00330; *p* = 0.00937), D-Arginine and D-ornithine metabolism (KEGG: dme00472; *p* = 0.01398), galactose metabolism (KEGG: dme00052; *p* = 0.04148) and histidine metabolism (KEGG: dme00340; *p* = 0.04379). The metabolic pathways, with over five different metabolites between *A. japonica*-parasitized and non-parasitized hosts, were as follows: purine metabolism, arginine biosynthesis, aminoacyl-tRNA biosynthesis, glycerophospholipid metabolism, ABC transporters, and arginine and proline metabolism. There were 10 differential metabolites in the pathway of purine metabolism, and all of them were upregulated in parasitized hosts (Table 1). In the pathways of aminoacyl-tRNA biosynthesis and ABC transporters, 83.33% (5/6) amino acids were upregulated, except L-aspartic acid, which was also enriched in the pathways of arginine biosynthesis, alanine, aspartate and glutamate metabolism and histidine metabolism. As for the pathway of glycerophospholipid metabolism, more than 60% (4/6) metabolites, including citicoline, glycerylphosphorylethanolamine, PC(16:0/16:0) and phosphocholine, were upregulated, while the contents of lysoPC(15:0) and PC(16:1(9Z)/0:0) in the pathway were downregulated. In addition, all the metabolites in the FoxO signaling pathway, mTOR signaling pathway and galactose metabolism, were increased in parasitized *Drosophila* hosts. As for the pathways including glutathione metabolism, arginine and proline metabolism and D-Arginine and D-ornithine metabolism, 75% (3/4), 60% (3/5) and 50% (1/2) of the metabolites were increased, respectively.

## 4. Discussion

It is well known that parasitoids need to change host metabolism to ensure the development and survival of their offspring; however, the underlying mechanisms are largely unknown [5,9]. Metabolomics analysis is extremely helpful to explore the molecular bases of host responses to parasitoid infection. Here, we compared metabolic profiling between *A. japonica*-parasitized and non-parasitized *D. melanogaster* hosts, and identified 3043 metabolites in the two groups of the host larvae. Among them, a total of 205 differential metabolites, consisting of 112 upregulated and 93 downregulated metabolites, were obtained, which belonged to 13 different metabolic pathways.

Lipids are widely distributed in insect individuals and are known to play important roles in growth and survival. Parasitoid wasps have evolved to modify host lipid metabolism and consume host fat as necessary nutrition, because they are incapable of synthesizing lipids [3,12,49,50]. Therefore, to some extent, lipid profiles are probably the best-characterized metabolic factor, of which the expression is associated with parasitoid infection [51,52]. In this study, *A. japonica* parasitism resulted in remarkable changes in host lipid metabolism. We found 63 lipids and lipid-like molecules changing their expression post parasitization, and more than 70% of these changed substances were downregulated, especially FAs, GPs and SPs. The decreased content of FAs may result in decreased activity of GP and SP metabolism, because FAs are building blocks of membrane lipids (e.g., GP and SP), as well as other nonpolar lipids with important storage functions [25]. Interestingly, in *Lysiphlebia japonica*-parasitized *Aphis gossypii* hosts, it has been reported that an increased abundance of FAs and GPs were detected [53,54]. Their findings were opposite to our results, indicating that different lipid manipulating strategies were taken in response to parasitizaion by different parasitoid species. SP, one of the necessary cellular lipid components, participates in multiple physiological activities, such as intercellular communication, cell proliferation and differentiation [55,56]. In 2017, Gao et al. have found that SPs were less abundant in the parasitized hosts, the cotton-melon aphid [54], which was similar to our results. In those differential FA lipids, we further found that almost all LCFAs and VLCFAs were declined in parasitized hosts, and almost all SFAs were upregulated, indicating that parasitoids require a high amount of SFAs, but not LCFAs and VLCFAs, from their hosts, which possibly benefits their development and reproduction.

Amino acids are one of the most important compositions of organic acids and derivatives, which are found to be crucial in egg-hatching [41]. Infection by parasitoid wasps usually leads to noticeable alterations in the levels of amino acids within their hosts. Our results confirmed the findings that 11 amino acids were increased in parasitized hosts, including L-histidine, L-proline, L-glutamate, L-tyrosine, L-arginine, argininosuccinic acid, gabapentin, 5-phosphonooxy-L-lysine, O-phosphohomoserine, L-2-Amino-5-(methylthio)pentanoic acid and aminoadipic acid. In addition, we also found levels of four amino acids were decreased, including O-phosphotyrosine, L-ornithine, L-aspartic acid and L-glutamate 5-semialdehyde. It has been reported that L-histidine and L-glutamate are momentous in the transition of insect development and metamorphosis [57]. L-proline is an endogenous neutral amino acid, which is essential for physiological and behavioral actions in the nervous system of vertebrates [58,59]. It has also been reported that L-arginine, argininosuccinic acid and aminoadipic acid can help insects synthesize immune effectors to participate in the immune priming response [60]. As such, our results suggested that parasitoids might need high amounts of L-histidine, L-glutamate and L-proline, to trigger their metamorphosis and neuronal development, along with high amounts of amino acids such as L-arginine, argininosuccinic acid and aminoadipic acid, to enhance their immunity. Moreover, previous studies have found that the content of protein amino acids (e.g., L-glutamate and L-histidine) were increased in hosts due to *Dastarcus helophoroides* or *Aphidius ervi* parasitization [61,62], which was consistent with our findings. Previous studies also showed that a reduction of tyrosine concentration was observed in *Cotesia congregata*-parasitized *Manduca sexta* host larvae and *Toxoneuron nigriceps*-parasitized *Heliothis virescens* host larvae [41,63]. However, the content of tyrosine was identified to be higher in host larvae post *A. japonica* parasitization. Thus, it is urgently necessary to identify the key elements that are in charge of the expression of amino acids within parasitized hosts, and to investigate the important functions of the amino acids in different parasitoid–host interaction.

Carbohydrates are the primary energy sources for longevity and fecundity of parasitoids. The principal carbohydrate sources available to parasitoid adults are nectar and honeydew, which contain abundant sucrose and other prevalent sugars [64,65,66]. Moreover, the host sugars are also essential for parasitoids to complete the developmental stages of their juveniles. For example, as the main storage sugar in insects, trehalose is used to generate glucose for glycolysis [67], and previous studies have shown that parasitism largely alters the level of trehalose and glucose in hosts [15,68,69,70]. In our study, sugar was also the most important part of the organic oxygen compounds in the changed metabolites. However, both the contents of trehalose and glucose were not significantly changed in *A. japonica*-parasitized host larvae at the first 3 h infection (Table S1). Nevertheless, we found that the majority of other sugars were upregulated in parasitized host larvae, which included three monosaccharides (D-erythro-D-galacto-octitol, galactaric acid and D-glucuronic acid), three disaccharides (D-maltose, sucrose and levan) and six oligosaccharides (maltohexaose, maltopentaose, verbascose, 3-galactosyllactose, mannan and stachyose). Previous reports have shown that the sugar nutrients can promote longevity, fecundity and population sizes of the insects [71,72]. In addition, three saccharides (levan, mannan and stachyose) were found to be involved in the immune responses [42,43,44]. Based on these data, we propose that the increased sugar metabolites are possibly beneficial to parasitoid embryonic and postembryonic development, and/or manipulate host immunity.

Parasites have great abilities to change host behaviors by manipulating neuromodulatory systems [45,46]. The behavioral responses are fascinating and varied, including suppression of host feeding behavior by increasing the octopamine level [47], and a transient paralysis of the host by blocking both acetylcholine and GABA, that mediate synaptic transmissions [48]. In *A. japonica*-parasitized host larvae, we also found changes in 10 neuromodulators, which belonged to four groups of metabolites (e.g., lipids and lipid-like molecules, organic acids and derivatives, nucleosides, nucleotides, and analogues, and benzenoids). The 10 neuromodulators were L-acetylcarnitine, gabapentin, L-glutamate, L-histidine, L-tyrosine, L-arginine, L-ornithine, L-aspartic acid, citicoline and felbamate. It has been reported that parasitism by *A. japonica* could cause a transient paralysis of the hosts [73]. Thus, it is possible that these differential neuromodulators are responsible to the behavioral regulation post *A. japonica* parasitization.

Endoparasitoids and ectoparasitoids are two kinds of parasitic insects with completely different egg-laying strategies. Ectoparasitoids lay their eggs on host integument; meanwhile, endoparasitoids exploit hosts by injecting egg(s) into their bodies. *A. japonica* is a typical larval-pupal endoparasitoid, and can successfully parasitize a large number of species across the *Drosophila* genus, including *D. suzukii*, an invasive pest insect [30,74,75]. To manipulate the host metabolism, parasitoids usually inject several parasitic factors along with the oviposition, which include venom, polydnaviruses and virus-like particles [5,8,33,76]. So far, the mechanisms of these parasitic factors regulating host metabolism are largely unclear. Since venom is the only reported parasitic factor of *A. japonica*, it is thus necessary to identify the venom composition in future studies, which will help dissect the underlying mechanisms.

## 5. Conclusions

Our findings provide a useful resource for understanding how parasitoids regulate the metabolism status of infected hosts. These results will broaden our knowledge on the nutritional requirements for the development and survival of parasitoids, and contribute to develop advanced pest-management strategies.

## Figures and Tables

**Table 1:** Significantly enriched metabolic pathways, identified in different metabolites.

Pathway	Metabolites	Regulation	*p*-Value	VIP
Purine metabolism (*p* = 2.51 × 10^−6^)	Adenosine 3’-monophosphate	up	9.05 × 10^−5^	5.54
	Adenosine monophosphate	up	1.21 × 10^−3^	3.33
	Adenylsuccinic acid	up	4.35 × 10^−4^	1.51
	ADP	up	6.31 × 10^−3^	1.02
	Guanosine	up	2.57 × 10^−2^	1.16
	Guanosine monophosphate	up	6.34 × 10^−3^	1.50
	Hypoxanthine	up	3.33 × 10^−3^	2.07
	Inosine	up	1.65 × 10^−3^	2.37
	Inosinic acid	up	7.76 × 10^−6^	3.61
	Uric acid	up	1.31 × 10^−2^	2.54
Arginine biosynthesis (*p* = 3 × 10^−5^)	Argininosuccinic acid	up	9.89 × 10^−3^	1.12
	L-Arginine	up	2.51 × 10^−6^	1.48
	L-Aspartic Acid	down	4.18 × 10^−4^	1.15
	L-Glutamate	up	7.75 × 10^−3^	2.50
	L-Ornithine	down	1.01 × 10^−5^	1.50
FoxO signaling pathway (*p* = 4.44 × 10^−5^)	L-Glutamate	up	7.75 × 10^−3^	2.50
	Adenosine monophosphate	up	1.21 × 10^−3^	3.33
	ADP	up	6.31 × 10^−3^	1.02
Aminoacyl-tRNA biosynthesis (*p* = 0.00019)	L-Arginine	up	2.51 × 10^−6^	1.48
	L-Aspartic Acid	down	4.18 × 10^−4^	1.15
	L-Glutamate	up	7.75 × 10^−3^	2.50
	L-Histidine	up	1.42 × 10^−5^	4.66
	L-Proline	up	5.07 × 10^−5^	5.37
	L-Tyrosine	up	3.09 × 10^−2^	1.36
Alanine, aspartate and glutamate metabolism (*p* = 0.00110)	Adenylsuccinic acid	up	4.35 × 10^−4^	1.51
	Argininosuccinic acid	up	9.89 × 10^−3^	1.12
	L-Aspartic Acid	down	4.18 × 10^−4^	1.15
	L-Glutamate	up	7.75 × 10^−3^	2.50
Glycerophospholipid metabolism (*p* = 0.00160)	Citicoline	up	5.49 × 10^−3^	1.04
	Glycerylphosphorylethanolamine	up	2.05 × 10^−5^	2.22
	LysoPC(15:0)	down	9.43 × 10^−3^	1.04
	PC(16:1(9Z)/0:0)	down	9.97 × 10^−3^	6.62
	PC(16:0/16:0)	up	1.73 × 10^−3^	5.91
	Phosphocholine	up	1.50 × 10^−3^	6.53
mTOR signaling pathway (*p* = 0.00165)	L-Arginine	up	2.51 × 10^−6^	1.48
	Adenosine monophosphate	up	1.21 × 10^−3^	3.33
Glutathione metabolism (*p* = 0.00349)	gamma-Glutamylalanine	up	6.73 × 10^−4^	1.13
	gamma-Glutamylcysteine	up	3.95 × 10^−3^	1.12
	L-Glutamate	up	7.75 × 10^−3^	2.50
	L-Ornithine	down	1.01 × 10^−5^	1.50
ABC transporters (*p* = 0.00426)	D-Maltose	up	1.78 × 10^−2^	8.29
	L-Arginine	up	2.51 × 10^−6^	1.48
	L-Aspartic Acid	down	4.18 × 10^−4^	1.15
	L-Glutamate	up	7.75 × 10^−3^	2.50
	L-Histidine	up	1.42 × 10^−5^	4.66
	Sucrose	up	1.17 × 10^−3^	10.22
Arginine and proline metabolism (*p* = 0.00937)	L-Arginine	up	2.51 × 10^−6^	1.48
	L-Glutamate	up	7.75 × 10^−3^	2.50
	L-Glutamate 5-semialdehyde	down	9.51 × 10^−3^	1.04
	L-Ornithine	down	1.01 × 10^−5^	1.50
	L-Proline	up	5.07 × 10^−5^	5.37
D-Arginine and D-ornithine metabolism (*p* = 0.01398)	L-Arginine	up	2.51 × 10^−6^	1.48
	L-Ornithine	down	1.01 × 10^−5^	1.50
Galactose metabolism (*p* = 0.04148)	Stachyose	up	3.34 × 10^−2^	2.63
	Sucrose	up	1.17 × 10^−3^	10.22
	UDP-D-galactose	up	2.70 × 10^−2^	1.39
Histidine metabolism (*p* = 0.04379)	L-Aspartic Acid	down	4.18 × 10^−4^	1.15
	L-Glutamate	up	7.75 × 10^−3^	2.50
	L-Histidine	up	1.42 × 10^−5^	4.66

## Data Availability

The metabolomics data have been deposited in the MetaboLights with accession number MTBLS6193. All data presented in this study are available in the article and supplementary material.

## Supplementary Materials

The following supporting information can be downloaded at: https://www.mdpi.com/article/10.3390/metabo13030336/s1, Table S1: Different metabolites between *A. japonica*-parasitized and nonparasitized hosts; Table S2: KEGG classification of metabolites in *A. japonica*-parasitized or non-parasitized hosts.
